# Spatial correlation between producer services agglomeration and carbon emissions in the Yangtze River Economic Belt based on point-of-interest

**DOI:** 10.1038/s41598-023-32803-1

**Published:** 2023-04-05

**Authors:** Peng Zeng, Lingjie Shang, Mengkun Xing

**Affiliations:** 1 School of Ethnology and Sociology, Guangxi University for Nationalities, Nanning, 530006 China; 2 School of Economics, Guangxi University for Nationalities, Nanning, 530006 China

**Keywords:** Environmental sciences, Environmental social sciences

## Abstract

Agglomeration of the industry significantly impacts economic performance and environmental sustainability. In line with its strategic context of striving to achieve carbon reduction targets, China is making efforts to optimize the producer services landscape to reduce carbon emissions. Understanding the spatial correlation between industrial agglomeration and carbon emissions is particularly crucial against this background. Based on POI and remote sensing data of China’s Yangtze River Economic Belt (YREB), the paper adopts the mean nearest neighbor analysis, kernel density analysis, and standard deviation ellipse to portray the agglomeration of producer services. Then uses Moran's *I* to present the spatial distribution characteristics of carbon emissions. Accordingly, the spatial heterogeneity of producer services agglomeration and carbon emissions is showed using the Geographic detector so as to provide strong support for industrial structure optimization and sustainable development. Here are some of the conclusions drawn from the study: (1) Producer services are a significant state of agglomeration in the provincial capitals and some central cities, with similar agglomeration patterns. (2) Carbon emissions exhibits significant spatial aggregation characteristics, with the spatial distribution pattern of "High west–Low east". (3) Wholesale and retail services industry is the primary risk factor that causes spatial differentiation of carbon emission intensity, "leasing and business services industry-wholesale and retail services industry" is the key interaction factor of the spatial differentiation. (4) Carbon emissions shows a downward trend followed by an upward trend as producer services agglomeration increases.

## Introduction

China has experienced an extensive transition since the reform and opening up^1^. There is no question that China has achieved world-renowned achievements. While developing its economy, China also attaches great importance to the issue of climate change. Optimizing the industrial structure is an effective way to reduce carbon emissions. Industrial agglomeration, as a form of industrial structure change, has a significant role in reducing carbon emissions^2^. There are several typical characteristics of producer services, such as knowledge-intensive, low pollution, and low consumption^3^. In order to adapt to China's "new era" of optimizing its industrial structure, producer services must be developed as quickly as possible. The Chinese government introduces several policies in an effort to develop the producer services, and reduce carbon dioxide emissions vigorously. In this vein, the YREB is the main battlefield where China's green development takes place, as well as the primary force behind its high-quality economic development. There should be a focus on whether producer services are clustered and how producer services agglomeration affects carbon emissions.

Research on the relationship between economic development and environmental pollution is the foundation for research on industrial agglomeration and environmental pollution^4^. In 1992, the environmental Kuznets curve, proposed by Grossman and Krueger, becomes the theoretical basis for the research^5^. The environmental Kuznets curve argues that increased income causes increased environmental pollution. However, the income will alleviate environmental pollution when severe to a certain extent^6,7^. Since then, many experts have studied the environmental Kuznets curve extensively. Fethi and Rahuma have verified the existence of it^8,9^. As an important economic development model, the industrial agglomeration has a scale effect and technology spillover effect^10^. The environmental effects of industrial agglomeration are mainly realized through externalities, which have been elaborated by rich theories^11^. The externality theory represented by Marshall believes that industrial agglomeration has obvious economic externalities and makes industrial agglomeration maintain competitive advantage^12^. With the development of theory, Krugman and others come up with the new economic geography theory, which believes that the technology spillover brought by industrial agglomeration is the main driving force for the sustainable development of industrial agglomeration, which greatly accelerates the process of cleaner production technology development^13^. Industrial agglomeration shows positive environmental externalities through technology spillovers. It effectively relieves environmental pressure. From the perspective of economies of scale theory, enterprises can reduce transaction costs and pollution control through economies of scale through industrial agglomeration^14^. This has paved the way for subsequent studies on the relationship between industrial agglomeration, producer services agglomeration, and environmental pollution. At present, several studies have examined it domestically and internationally. In terms of content, many scholars analyze industrial agglomeration externalities on the environment, examined the space autocorrelation^15,16^ and the spatial spillover effects^17,18^. Regional carbon emissions are affected by industrial agglomeration but not those in surrounding cities. Or surrounding carbon emissions are impacted but not those in regional cities^19–21^. In terms of methodology, scholars have evaluated industrial agglomeration by calculating the location quotient index and Gini coefficient^22,23^. The agglomeration of producer services is evaluated by calculating the producer services agglomeration index^24^. Meanwhile, making use of the spatial Durbin model, dynamic GMM model, and STIRPAT model to reflect how industrial agglomeration and producer services agglomeration impact environmental pollution in Chinese cities^25–27^. The study area covers specific provinces, cities, regions, etc.^28^. Different research methods, data sources, and study regions have led to different findings. The scholarly opinion on how industrial agglomeration affects environmental pollution can be classified into three categories. First, industrial agglomeration will be enough to mitigate environmental pollution and improve the environment's ecological quality. It is expected that industrial agglomeration will have a negative effect on pollution^29^. The synergistic agglomeration of producer services and manufacturing industries has a significant contribution to ecological and environmental management^30^. With the development of China's economy, the scale of foreign direct investment (FDI) is gradually expanding. Agglomeration of industrial activities and FDI coexist in China's economic development^31^. Therefore, it needs to pay attention to FDI. Shijie finds that industrial agglomeration has an enhanced environmental improvement influence when foreign direct investment is present^32^. Second, industrial agglomeration increases carbon emissions and intensifies environmental pollution^33,34^. While industrial agglomeration will increase industrial efficiency, it does not reduce carbon emissions as expected and causes serious environmental pollution^35,36^. Andersson finds that industrial agglomeration will aggravate ecological degradation due to the existence of the scale effect^37^. Since the deepening of research, local governments' competitive behavior has been fully considered in the study. Compared with industrial agglomeration alone, the crowding and concentrated emission effect brought by industrial agglomeration and the local government's competitive behavior can exacerbate environmental pollution^38^. Third, the relationship between industrial agglomeration and environmental pollution is nonlinear^39,40^. Zhu and Xia test whether industrial agglomeration and pollution were related to different levels of urbanization using a threshold model^41^. The results proved that they have a nonlinear inverted U-shaped structure rather than a simple linear relationship^42^. Additionally, it can be shown that industrial agglomerations are not linearly related to carbon emissions pollution once energy intensity and technological innovation are taken into consideration^43,44^. Consequently, the relationship is uncertain^45^. In studying of the relationship between industrial agglomeration and environmental pollution, some scholars have begun to pay close attention to the relationship between producer services agglomeration and environmental pollution. They usually analyze the impact of the environmental externalities of producer services agglomeration. Producer services are characterized by low energy consumption, low pollution emissions and, low carbon. Therefore, it is an important channel to develop a low-carbon economy and achieve green development. Existing researches generally find that producer services agglomeration can significantly reduce carbon emissions and have a spatial spillover effect^24^. It positively impacts carbon efficiency through the scale effect and technology spillover effect. In addition, producer services agglomeration can positively promote enterprises to enter the market^27^.

In summary, although the research methods and results on the relationship between producer services agglomeration and environmental pollution are mature and abundant, there are some shortcomings. Firstly, previous research has mainly relied on regional statistical data and panel data for its findings^46^. No research has used point-of-interest (POI) and remote sensing data. POI can map entity information to a virtual geographic space for abstract representation and show the spatial information of point elements in various social and economic areas^47^, which provides new ideas for this study. Secondly, most scholars focus on macro and micro levels. The research regions are Chinese provinces^48^ and cities^49^. Based on this, the paper chooses the YREB as the study area. It enriches the research perspective. Thirdly, previous literature mostly use spatial econometric models, which better deal with spatial correlation and spatial spillover effects but neglect to explore the spatial association between producer services agglomeration and carbon emissions. The research uses the geographic detector to detect spatial association. This paper adopts the mean nearest neighbor analysis, kernel density analysis, and standard deviation ellipse to portray the agglomeration characteristics of producer services, then uses Moran's *I* to present the spatial distribution characteristics of carbon emissions. Moreover, it uses the Geographic detector to show the spatial heterogeneity of producer services agglomeration and carbon emissions. In this way, it can not only make up for the shortcomings of related studies but also optimize the agglomeration pattern of producer services. The YREB will be developed into an economical, efficient, ecologically sound green, circular, and low-carbon ecological economy demonstration Belt. The YREB will realize high-quality development.

## Study design

### Research mechanism

**Figure 1:**
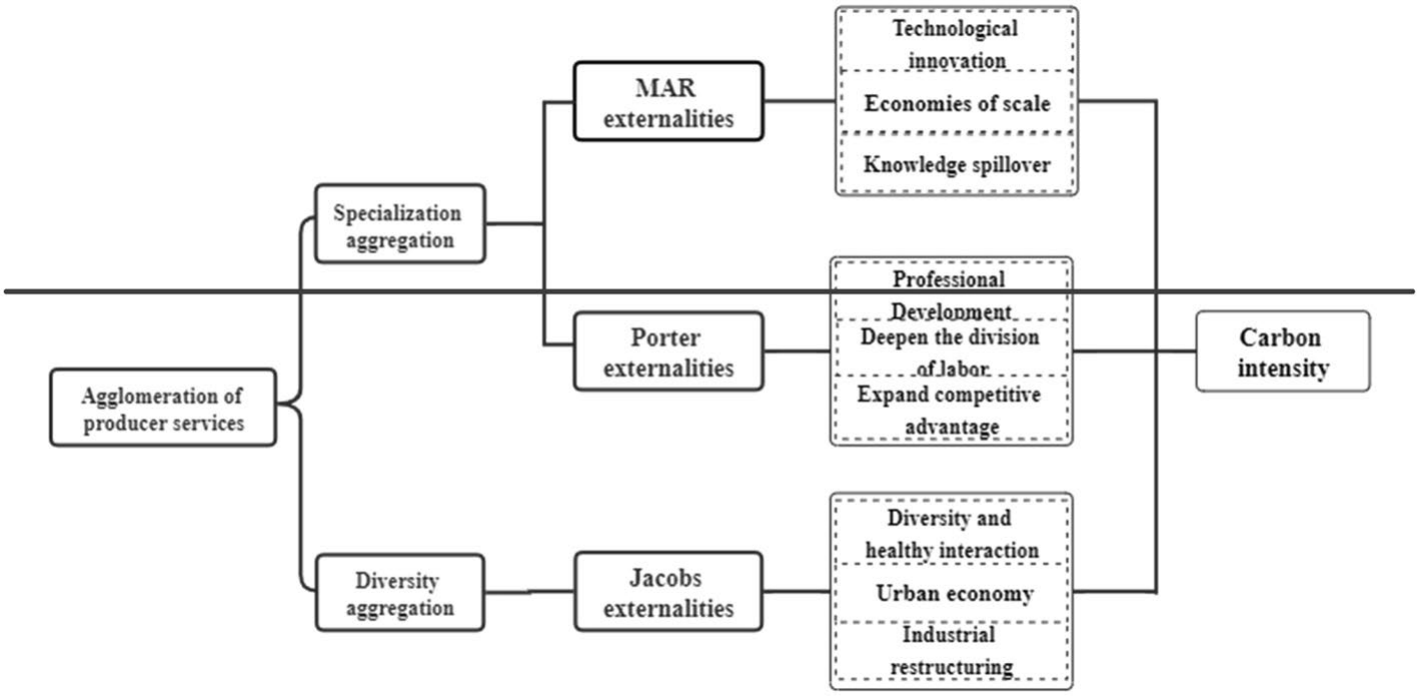
Mechanism of the effect of producer services agglomeration.

The environmental effects of industrial agglomeration are mainly realized through externalities. As one of the important bridges to introduce human capital, knowledge, and technology into goods and services, producer services can exert their environmental effects to influence carbon emissions through deepening MAR externalities, Jacobs’ externalities, and Porter externalities. MAR externalities argue that technological innovation can strengthen the specialization of enterprises and achieve better development through knowledge and technology spillover effects^50^. Jacobs’ externality focuses on the externalities of industries. The agglomeration between different market players stimulates the positive interaction between diverse sectors to obtain technological innovation in the intense collision of knowledge and human capital. Based on Porter's externalities, externalities originate from the interaction between competitive specialization and division of labor, and technological innovation. MAR externality and Porter externality are closely related to producer services specialization agglomeration, while Jacob’s externality is specific to producer services diversified agglomeration, which is shown in Fig. 1.

Producer services specialized agglomeration impact on carbon emissions. The concept of specialized agglomeration involves the development and growth of the same type of industry in a region. To exploit the MAR externality, enterprises in the producer service agglomeration area undertake technological innovation in order to obtain more revenue. By integrating input–output correlations, it is possible to integrate environmental protection and low-carbon technologies into the production process, thus increasing the industrial value chain^51^. Industrial value chain extension brings economies of scale and technological knowledge spillovers, resulting in a lower carbon footprint in the region. With the increasing level of specialized agglomeration, the same and different types of producer services gather to obtain the benefits of agglomeration. They engage in professional human capital exchange and cooperation. Additionally, they facilitate the flow and diffusion of technology. It is conducive to deepening and amplifying the competitive advantages of the cluster. Forming a competitive advantage will give rise to great requirements by deepening the division of the labor system, which will counteract producer services agglomeration and reduce carbon emissions^52^. It is a typical Porter externality.

Producer services diversified agglomeration impact on carbon emissions. Diversified agglomeration means the agglomeration of different industries in the same area and sharing the market. Diversified sectors gather in a region to promote industrial transformation and upgrading. It contributes to total factor productivity^30^. The degree of diversified industrial agglomeration and the scale of cities effectively match each other. Carbon emissions can be reduced by their positive interactions. Firstly, producer services diversified agglomeration can shorten the transportation distance and reduce the cost of transactions. Secondly, it can help highly adaptive specialized talents and knowledge exchange across industries to achieve collaborative technological innovation. It is necessary to form a benign mechanism of division of labor and collaboration, which will have a suppressive effect on carbon emissions.

### Study area and data sources

#### Study area

**Figure 2:**
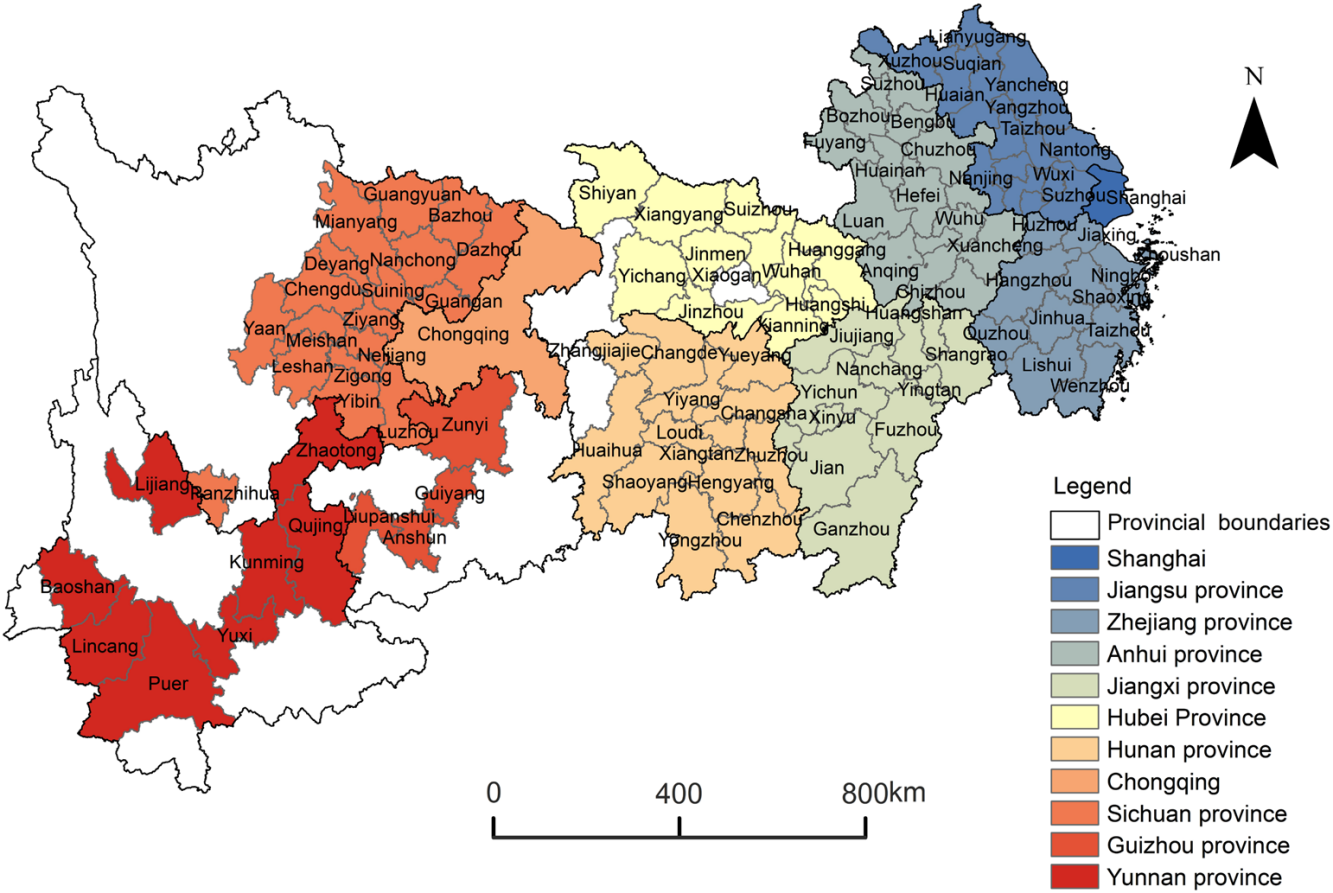
Research area.

The YREB includes Shanghai, Anhui, Jiangxi, Jiangsu, Hubei, Guizhou, Zhejiang, Hunan, Yunnan, Sichuan, and Chongqing. It has a total area of 2,057,000 km^2^ and three major urban agglomerations. Known as the YREB, it spans China's three major economic zones, East-Central-West, and is the one of most significant strategic development areas. In 2021, the YREB accounts for 46.36% of the national economic output, with an average growth rate of 8.7%. As one of the "three major support belts" for China's regional development, it contributes to China's economic and social development. It’s also helpful for achieving China's carbon emissions reduction goals. Therefore, the paper chooses 108 cities in the YREB as its scale range, which is shown in Fig. 2.

#### Data sources

Big Data. First, regarding the industry division standard of the National Economic Industry Classification (GB/T 4754-2017) and the Statistical Classification of Producer Services (2019), the producer services are defined as 53 industry subcategories in six industry categories, including finance and insurance, real estate, leasing and business services, scientific research and technical services, wholesale and retail, and transportation, storage, and postal services (Table 1). Then, EasyPoi software (http://www.wupaas.com/) crawls the POI data of producer service industries in November 2021 on the AutoNavi Map. To maintain that the data is accurate and reliable, a total of 6,661,900 points of interest are obtained through the pre-processing process of spatial matching and de-duplication.

**Table 1:** Classification of producer services based on POI data.

Industry classification	Quantity/million	Content of POI data
Finance and Insurance industry	24.68	Banks, securities companies, insurance companies, finance companies, futures companies
Real Estate industry	47.81	Business office buildings, residential communities, sales centers, real estate agents, property companies
Leasing and Business Service industry	5.04	Automobile leasing companies, machinery leasing companies, daily necessities leasing companies, travel agencies, advertising companies, law firms, accounting firms, appraisal firms, certification firms, talent markets
Scientific Research and Technology Service industry	9.71	Research institutes, research institutes, experimental centers, laboratories, photographic expansion stores
Wholesale and retail service industry	552.28	Shopping malls, supermarkets, convenience stores, home appliance stores, pharmacies, gas stations, filling stations, charging stations, flower, bird, fish, and insect markets, home building material cities, general markets, sports, and cultural goods stores, clothing, shoes, and leather goods stores, specialty stores, outlet stores, cosmetics stores, car sales, motorcycle sales, auto, and motorcycle parts sales, pharmaceutical and medical equipment wholesale
Transportation, storage, and postal industry	26.76	Bus stations, train stations, subway stations, airports, transportation ticket sales outlets, post offices, logistics couriers, logistics storage sites

Remote sensing data. The carbon emissions data in 2021 are obtained from the study of^53^. The first step of this study is to integrate the different satellite observation data from GOSAT and OCO-2 into a long-sequence XCO_2_ data set with a unified time and space scale. Then, using the Spatio-temporal kriging method, the XCO_2_ value of the midline point of the 1° grid is interpolated to produce the global 1° space–time continuous XCO_2_. The paper uses the inverse distance weight method of interpolation sampling to obtain the carbon emissions data to overcome the problem of high resolution. The unit for the carbon emissions data in 2021 is kilogram per m^3^.

### Research methodology

#### Mean nearest neighbor analysis

Mean nearest neighbor analysis is a method to calculate the average of the nearest distance between point elements. It also measures the degree of agglomeration and dispersion of data at different points^54^. This paper adopts it to detect whether the producer services are clustered and compare the degree of clustering of each producer services. When the nearest neighbor distance index (NNI) is less than 1, it indicates an agglomeration. Agglomeration is higher when NNI is smaller. When NNI exceeds 1, it means a discrete distance. The larger NNI is, the higher the degree of dispersion is. When NNI approaches 1, the probability of a random distribution increases. Here is the formula:

1 $$ NNI = \frac{{\frac{1}{n}\mathop \sum \nolimits_{i = 1}^{n} d_{i} }}{{0.5*\sqrt {A/n} }} $$

*A* is the city, *d*_*i*_ is the average distance between each urban element and its proximity, and *n* is the sample size.

#### Kernel density analysis method

The method is widely applied in spatial analysis of point data to estimate the density of POI and generates continuous spatial distribution results^55^. This paper uses it to visualize six types of producer services, which directly reflects the spatial distribution characteristics. The higher the kernel density value is, the higher the concentration of point data distribution is. Following is the formula:

2 $$ H_{n} (x) = \frac{1}{nh}\mathop \sum \limits_{i = 1}^{n} f\left( {\frac{{x - x_{i} }}{r}} \right) $$

*x* denotes the location of the element, *h* denotes the radius of the circular region formed by $x$ as the center, *n* denotes the number of samples, *f* denotes the kernel function, and *x*_*i*_ denotes the specific location in the space of the producer services in the circular region formed by *x* as the center.

#### Standard deviation ellipse method

The method can directly demonstrate the clustering trend, the core range of distribution, and the direction of distribution of producer services in the YREB^56^.

Rotation angle:

3 $$ \tan \theta = \frac{{\mathop \sum \nolimits_{i = 1}^{n} \overline{{x_{i} }}^{2} - \mathop \sum \nolimits_{i = 1}^{n} \overline{{y_{i} }}^{2} + \sqrt {\left( {\mathop \sum \nolimits_{i = 1}^{n} \overline{{x_{i} }}^{2} - \mathop \sum \nolimits_{i = 1}^{n} \overline{{y_{i} }}^{2} } \right)^{2} + 4\left( {\sum \overline{{x_{i} }} \overline{{y_{i} }} } \right)^{2} } }}{{2\left( {\sum \overline{{x_{i} }} \overline{{y_{i} }} } \right)^{2} }} $$

$\theta$ is the azimuth of the ellipse, $\overline{{x }_{i}}$ and $\overline{{y }_{i}}$ denote the coordinate deviation of POI, $n$ is the study objects’ number.

Standard deviation distance of axis length:

4 $$ \sigma_{x} = \sqrt {\frac{{\mathop \sum \nolimits_{i = 1}^{n} \left( {x_{i} - \overline{x}} \right)}}{n}} $$

5 $$ \sigma_{y} = \sqrt {\frac{{\mathop \sum \nolimits_{i = 1}^{n} \left( {y_{i} - \overline{y}} \right)}}{n}} $$

$\sigma_{x}$ and $\sigma_{y}$ denote the lengths of the x-axis and y-axis, respectively. $\overline{x}$ and $\overline{y}$ represent all elements’ average center.

#### Spatial autocorrelation analysis

1. Global spatial autocorrelation examines the attribute values of spatially adjacent area cells^57^. The research adopts the global Moran' *I* to reflect the overall characteristics of carbon emissions^58^. Based on the following calculation:
  6 $$ I = \frac{{n\mathop \sum \nolimits_{i = 1}^{n} \mathop \sum \nolimits_{j = 1}^{n} W_{ij} \left( {x_{i} - \overline{x}} \right)\left( {x_{j} - \overline{x}} \right)}}{{\mathop \sum \nolimits_{i = 1}^{n} \mathop \sum \nolimits_{j = 1}^{n} W_{ij} \mathop \sum \nolimits_{i = 1}^{n} \left( {x_{j} - \overline{x}} \right)}} $$
  $$I $$ is the global spatial autocorrelation index. $$\overline{x }$$ indicates the average value, $$x_{i}$$ and $$x_{j}$$ denote the carbon emissions, *n* denotes the number of prefecture-level cities.
2. Local spatial autocorrelation, which reveals the correlation degree between an attribute of a spatial unit and its neighboring units, can clearly indicate the location of spatial agglomeration or dispersion. Local spatial autocorrelation is generally reflected by the Moran scatter diagram first proposed by^59^, including four forms of spatial linkage. To better reflect the local autocorrelation, the spatial distribution graph is added in this paper^60^.

#### Geographic detector

A key assumption of the geographic detector is that if an independent variable influences a dependent variable, the independent variable and the dependent variable should have similar spatial characteristics^61^. This paper uses factor detectors, interactive factor detectors, and risk detectors to study the influence of producer services on carbon emissions in the YREB from the perspective of spatial heterogeneity.

1. Factor detection, which detects the extent to which each sector of the producer services industry explains the spatial heterogeneity of carbon emission intensity. Q-values are used to express the result. Based on the following calculation:
  7 $$ q_{X,Y} = 1 - \frac{{\mathop \sum \nolimits_{i = 1}^{n} N_{X,i} \sigma_{{Y_{X,i} }}^{2} }}{{N\sigma_{Y}^{2} }} $$
  $$q_{X,Y} $$ is the explanatory power of $$X$$ on $$Y$$. $$X$$ is producer services, $$ Y$$ is carbon emissions, $$N_{X,i }$$ is the sample number. $$n$$ is the number of cities. $$\sigma_{Y}^{2}$$ is the discrete variance of the carbon emissions, $$\sigma_{{Y_{X,i} }}^{2}$$ is the discrete variance of a city's carbon emissions. A larger *q* indicates that stronger explanatory power of producer services on the spatial differentiation of carbon emissions and vice versa^62^.
2. Interaction detection, which assesses whether the explanatory power of X on Y is enhanced or weakened when the two factors act together. Calculating the q-value is the method of evaluating the interaction between two factors. As a final step, compare q(X_1_),q(X_2_), and q(X_1_ ∩ X_2_). Two independent variables can interact with the dependent variable in five different ways:
  I. If q(X_1_ ∩ X_2_) < min[q(X_1_),q(X_2_)], it means that the factors X_1_ and X_2_ are nonlinearly weakened after the interaction.
  II. If min[q(X_1_), q(X_2_)] < q(X_1_ ∩ X_2_ < max[q(X_1_), q(X_2_)], it means that X_1_ and X_2_ interact with each other with a single factor nonlinearly weakened.
  III. If q(X_1_ ∩ X_2_) > max[q(X_1_),q(X_2_)] and q(X_1_ ∩ X_2_) < q(X_1_) + q(X_2_), it means that X_1_ and X_2_ are bifactor-enhanced after the interaction.
  IV. If q(X_1_ ∩ X_2_) = q(X_1_) + q(X_2_), it means that X_1_ and X_2_ are independent of each other.
  V. If q(X_1_ ∩ X_2_) > q(X1) + q(X_2_), it means that X_1_ and X_2_ are nonlinearly enhanced after the interaction.
3. Risk detection is used to determine whether there is a significant difference in the mean carbon intensity of the two subregions.

## Results

Whether urban elements are spatially clustered and how they are clustered are the basic prerequisites for judging the spatial layout within a city. They are also important factors affecting the spatial structure within a city. Therefore, this section depicts the spatial distribution pattern of producer services and carbon emissions then explores the spatial association between producer services and carbon emissions.

### Spatial pattern of producer services in the YREB

#### Analysis of the spatial distribution pattern of producer services in the YREB

The closest proximity index of each industry in the producer services is calculated using ArcGIS10.8, developed by Environmental Systems Research Institute. The results are shown in Table 2. The range of the closest distance index is 0.0979–0.1862, which is less than 1. The Z-value is less than − 4055.63, which passes the test at 1% significance level. Significant agglomeration exists in all sectors of producer services.

**Table 2:** Nearest Distance Index for producer services.

Industry	Average nearest neighbor distance (m)	Desired nearest neighbor distance (m)	Nearest Neighbor Distance Index	Z-test value	General Characteristics
Finance and Insurance industry	211.35	1679.55	0.1258	− 830.81	Clustering
Real Estate industry	221.92	1195.33	0.1857	− 1077.18	Clustering
Leasing and Business Service industry	640.96	3642.36	0.1759	− 353.36	Clustering
Scientific Research and Technology Service industry	492.03	2662.03	0.1848	− 480.18	Clustering
Wholesale and retail service industry	34.95	356.92	0.0979	− 4055.63	Clustering
Transportation, storage and postal industry	301.21	1618.05	0.1862	− 805.35	Clustering

From the clustering degree of each industry in the producer services, the wholesale and retail service industry has the highest clustering degree. The average nearest neighbor distance of the financial and insurance industry samples is 211.35 m, indicating a high average density. NNI is 0.1258, suggesting that it is highly concentrated in local areas. The YREB is one of the most active regions in China in terms of economic development. As one of the critical features of sustainable urban development, financial agglomeration can generate external scale effects and support development^63^. The average nearest neighbor distance of the real estate industry is 221.92 m, and NNI is 0.1857, indicating that the real estate industry is not widely but more uniformly distributed. The average nearest neighbor distance of the leasing and business service industry is 640.96 m, and NNI is 0.1759, indicating that it is widely but less uniformly distributed. The average nearest neighbor distance of the scientific research and technical service industry is 492.03 m, and NNI is 0.1848, indicating that it is widely distributed but not quite uniform. The average closest distance of the wholesale and retail service industry is 34.95 m, and NNI is 0.0979, indicating that it is not widely distributed and not uniform, which is consistent with the highest degree of aggregation of the wholesale and retail service industry. The average closest distance of transportation, storage, and postal industry is 301.21 m, and NNI is 0.1862, which indicates that it is not widely distributed but more uniform.

#### Analysis of spatial aggregation characteristics of producer services in the* YREB*

**Figure 3:**
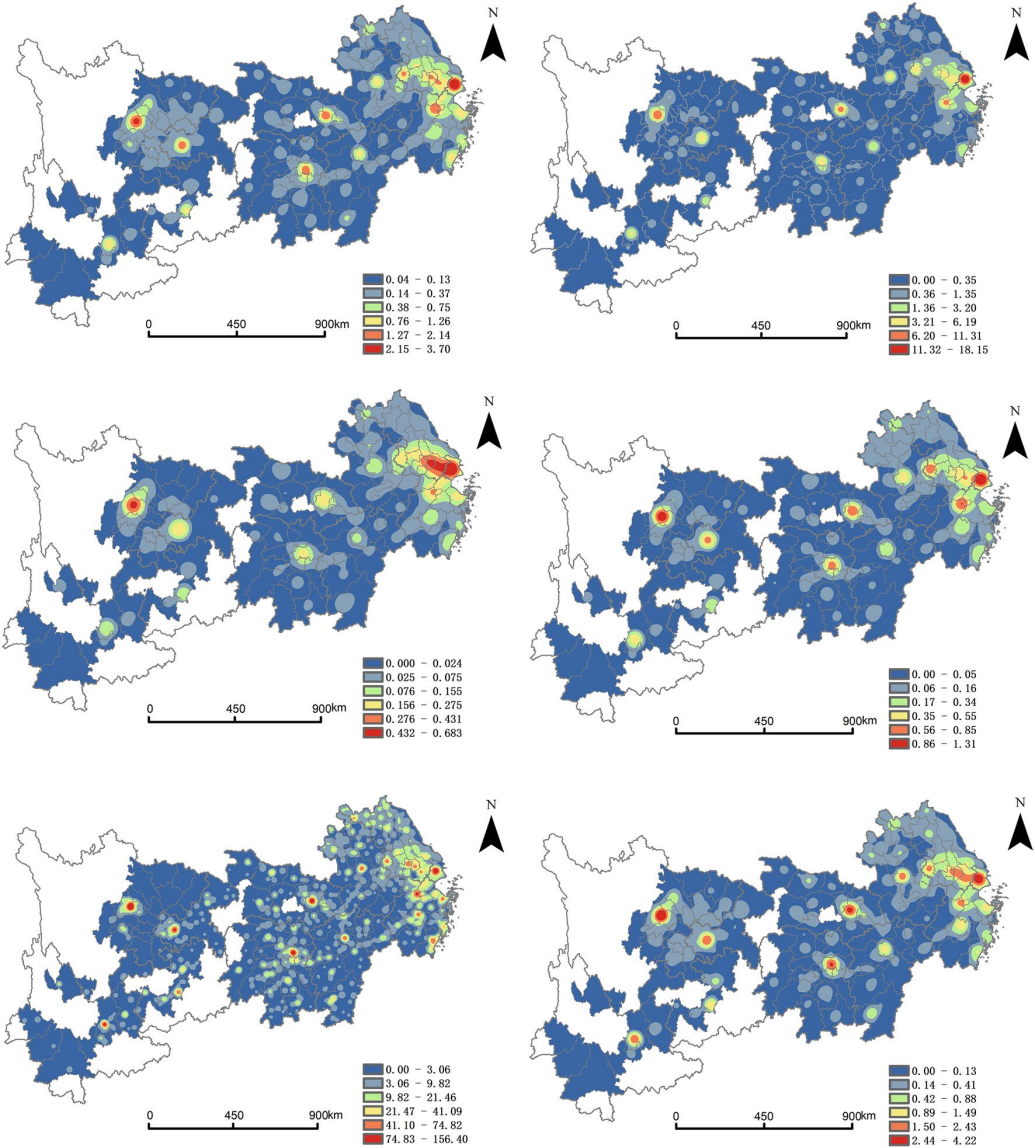
Kernel density of producer service.

This paper uses ArcGIS 10.8 to visualize the point of interest. In this paper, the kernel density is divided into six grades by using the natural subsection point method, and the kernel density of each grade is shown in the graph according to the gradient color. The nuclear density distribution of producer services is shown in Fig. 3, where the red area indicates the high concentration area of each industry in the producer services.

Financial and insurance industry. It is estimated that the peak core density for the financial and insurance industry is between 2.15 and 3.70 per square kilometer. The financial and insurance industry in the YREB presents a decentralized and combined spatial distribution pattern, which is in line with the multi-pole nuclear agglomeration pattern. The main concentration centers of the financial and insurance industry are located in ten cities, such as Shanghai and Suzhou, showing a "ten-center" spatial concentration structure. Seven small nuclei are formed in Hefei and Taizhou, etc. As the main pillar industry of the YREB, the financial and insurance industry in Shanghai, Jiangsu, and Zhejiang are more prominent. Improving the specialization level of the financial and insurance industry in the central city, can produce the agglomeration radiation effect on the surrounding cities.

Real estate industry. Its peak density is between 11.32 and 18.75 per square kilometer. The high-density agglomeration area shows a radial expansion-type aggregation pattern, spreading along the east-central-west direction. There are only four independent small polar cores formed in Shanghai, Hangzhou, Wuhan, and Chengdu. The real estate industry is one of the most concerned industries in producer services. Areas, with their development, resource advantages, and strategic planning, have a more diversified real estate industry pattern. It deserves our further attention.

Leasing and business service industry. Its peak core density of it is between 0.432 and 0.683 per square kilometer. The high-density agglomeration area shows the spatial pattern of concentrated clusters, in line with the distribution characteristics of "large clustering-small dispersal". The clustering centers in Shanghai and Wuxi form a continuous cluster, while the clustering in Chengdu is faceted. This may be related to the small number of POI in leasing and business services. In addition, Nanjing, Zhenjiang, and Wuhan have fragmented clusters. The spatial heterogeneity of the industry is not significant.

Scientific research and technical service industry. The peak nuclear density of the scientific research and technology service industry is between 0.86 and 1.31 per square kilometer. The high-density agglomeration area shows dispersed and combined spatial distribution patterns, in line with the agglomeration pattern of multi-polar nuclear clusters. Compared with other cities, central cities’ nucleus density of scientific research and technology service industry is higher. Talent is an important guarantee to promote economic and social development and a key factor in developing advanced productive forces. To provide richer talent and technology reserves, cities develop industries such as scientific and technological consultation and information technology to serve the manufacturing industry.

Wholesale and retail service industry. The peak nuclear density of the wholesale and retail service industry is between 74.83 and 156.40 per square kilometer. It shows a belt-like aggregation pattern extending outward from the high nuclear density area in the scale of the YREB. Eighteen high-density agglomerations are formed in cities such as Shanghai and Suzhou, where wholesale and retail industries are highly concentrated and spread to peripheral cities. In addition, sub-clustering centers are formed in 11 cities, such as Nantong City and Yangzhou City, which promote the polycentric development of the wholesale and retail service industry.

Transportation, storage, and postal industry. The peak nuclear density of transportation, storage, and postal industry is between 2.44 and 4.22 per square kilometer. It shows the agglomeration pattern of multi-pole nuclear clusters. The main clusters of it are distributed in the Yangtze River Delta region. It forms a chain of clusters spreading in Shanghai, Jiangsu, and Zhejiang. In Zhejiang Province, Hangzhou, Ningbo, and four other cities are agglomeration centers. This corresponds to the reality that the development of cross-border e-commerce in Zhejiang is in the leading position. This shows that the development of the transportation, storage, and postal industry has gradually lowered the barriers to cooperation between enterprises of the Yangtze River Delta Economic Zone, and the trend of cross-regional enterprises cooperation is obvious.

In short, the polycentric agglomeration phenomenon is most obvious and widely distributed in the wholesale and retail service industry, which may be related to the number of POI of the wholesale and retail service industry. Most agglomeration and sub-glomeration centers of each industry in the producer services are located in Chinese municipalities such as Shanghai and Chongqing and provincial capitals such as Nanjing and Hangzhou. This indicates that the producer services have the location preference of large cities with obvious economic, social and human capital pointers. The reason is closely related to the new development pattern of “an axis—two wings—three levels and multiple points” in the YREB. Among them, "an axis" plays a central role in Shanghai, Wuhan, and Chongqing; "two wings" refers primarily to the extension of the radiation-driving effect from the YREB's main axis, northern and southern, respectively. "Three poles" refer to the Yangtze River Delta, Triangle of Central China, ChengYu; "multi-point" refers to the support role of prefecture-level cities. With the development of social economy, the "point-axis" will undoubtedly develop into the "point-axis-colony". This is a typical "point-axis system" theory. The key to achieving the rise of the YREB is to play the role of agglomeration and radiation of producer services in central cities.

#### Analysis of the development trend of producer services clustering in the YREB

**Figure 4:**
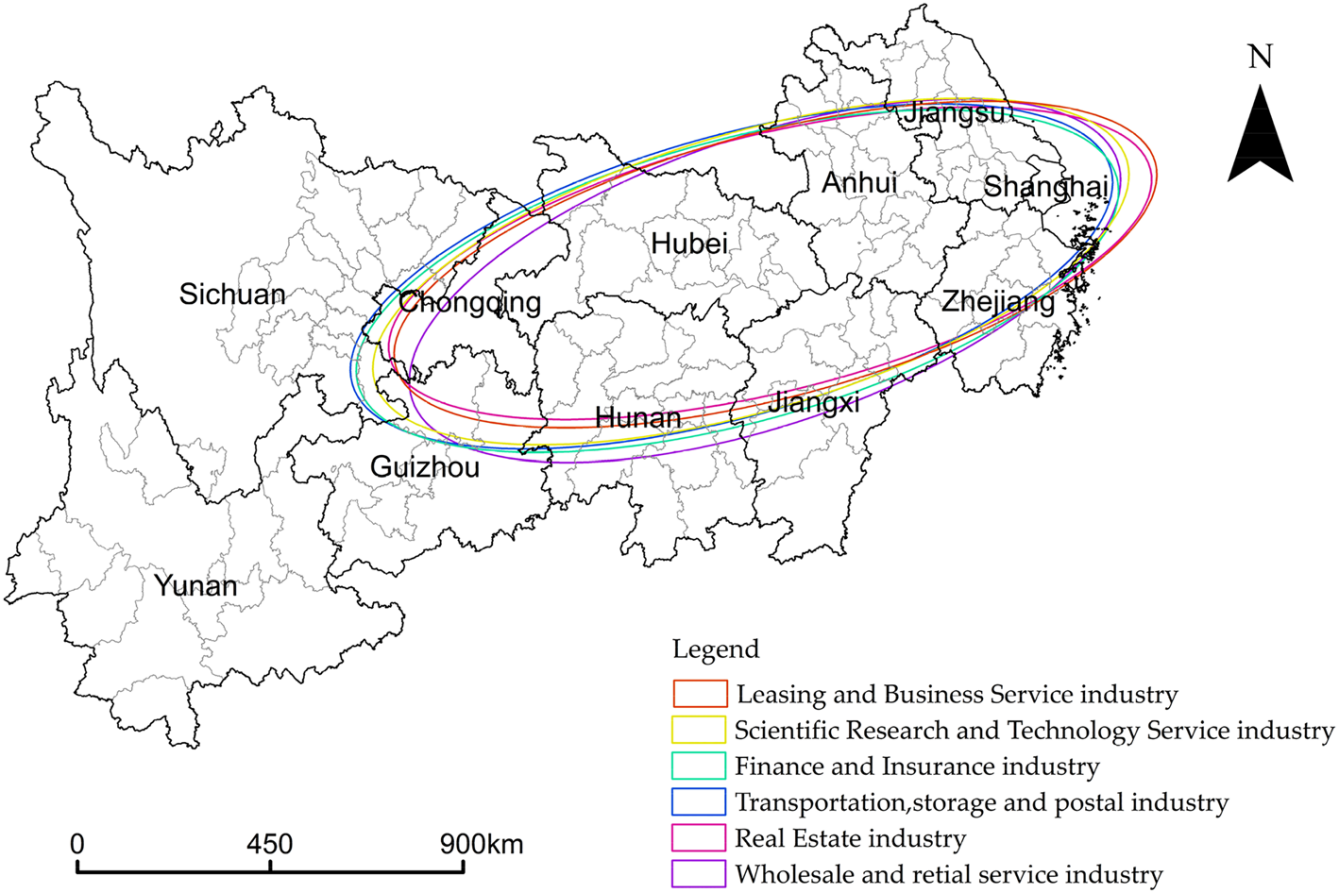
Standard deviation ellipse of producer services.

ArcGIS 10.8 is used to analyze the development trend of producer services agglomeration. The standard deviation ellipse parameter (Table 3) and standard deviation ellipse diagram (Fig. 4) is obtained. From the shape of the standard deviation ellipse, the producer services are distributed southwest-northeast, which points to the development axis of “Chongqing-Wuhan-Shanghai”. There is little difference in the shape of the six major industries. The oval area of the standard deviation of each industry does not show the phenomenon of "secondary industry dominance", which indicates that the simultaneous centrifugation has promoted the formation of a polycentric city structure under the radiation drive of the main axis of the Yangtze River. The X-axis of the transportation, storage, and postal industry is the longest at 871,280.42, which means that they have the most apparent distribution directions. The X-axis of the wholesale and retail service industry is the shortest at 823,074.13, with the least obvious distribution direction. The industry’s distribution is in the middle of these two. The Y-axis of the wholesale and retail service industry is the longest at 319,315.59. Despite its extensive distribution, the wholesale and retail service industry has the least obvious centripetal. Their distribution is the widest, but their centripetal is the smallest. The Y-axis of the real estate industry is the shortest at 284,046.08, indicating that its centrality of it is the most obvious. The deflection angle for the real estate industry is 76.03°, the highest of any industry. It shows that the shape of the YREB significantly impacts the agglomeration of the real estate industry. The deflection angle for the wholesale and retail service industry is 71.27°, the smallest of any industry. The deflection angles for the rest of the industry are in the middle of these two. From the perspective of development trend, the total number of spatial associations within the Yangtze River Delta urban agglomeration had increased from 108 at the beginning of the observation period to 133 at the end, with a maximum total of 650. Additionally, the overall network density has also increased from 0.166 to 0.205.

**Table 3:** Standard deviation ellipse parameters for each industry in the producer services.

Industry category	X-axis length	Y-axis length	Deflection angle θ (°)
Finance and Insurance industry	869,308.07	312,295.14	74.69
Real Estate industry	865,970.65	284,046.08	76.03
Leasing and Business Service industry	869,300.48	292,217.46	74.99
Scientific Research and Technology Service industry	866,690.28	306,445.09	73.52
Wholesale and retail service industry	823,074.13	319,315.59	71.27
Transportation, storage and postal industry	871,280.42	309,684.42	74.14

### Spatial patterns of carbon emissions in the YREB

#### Analysis of the spatial distribution characteristics of carbon emissions in the YREB

**Figure 5:**
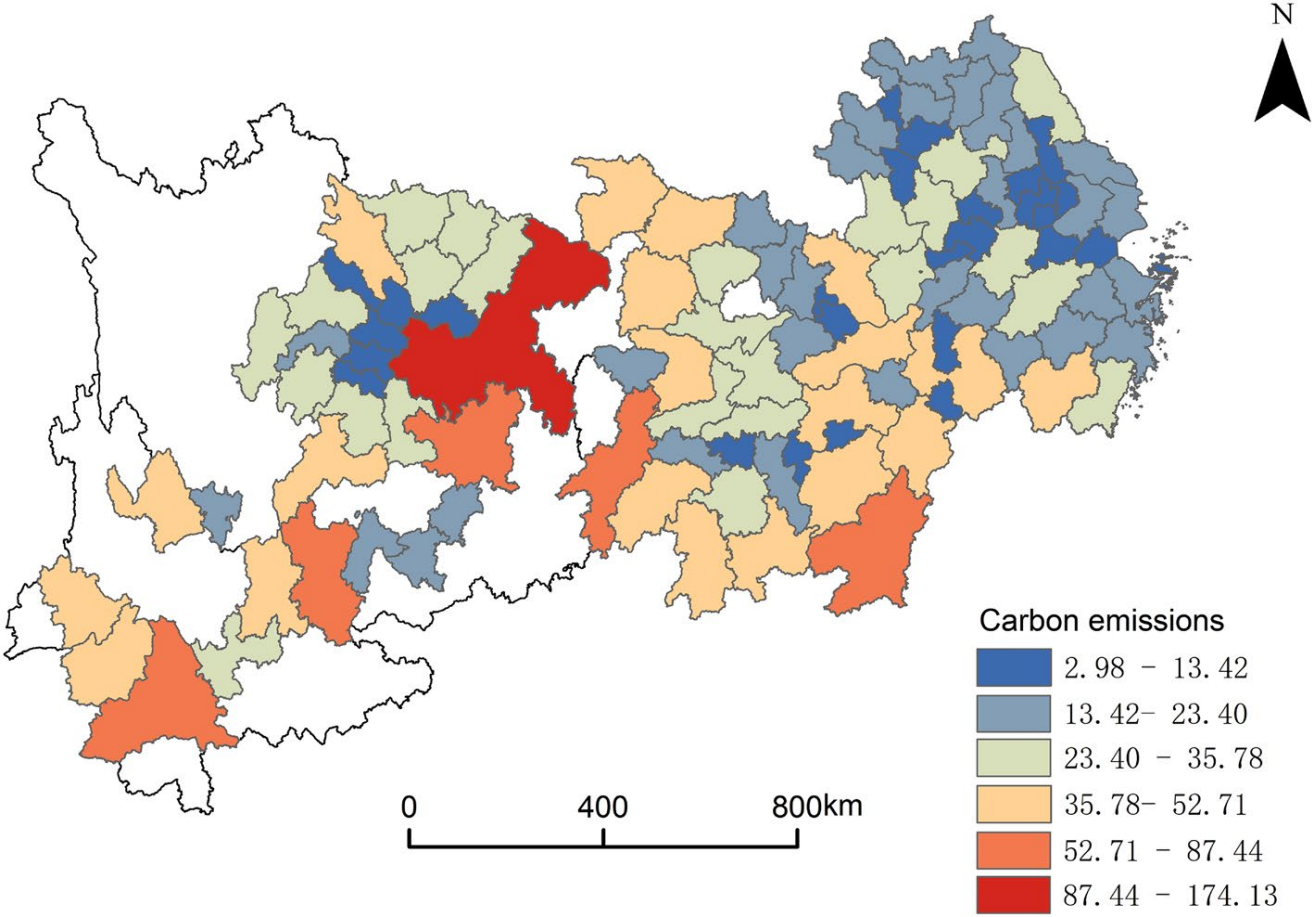
Current status of carbon emissions.

The carbon emissions data were imported into ArcGIS10.8. The research uses the natural discontinuous point classification method to classify the carbon emissions into six levels and exports. The results are shown in Fig. 5. Concerning carbon emission intensity, the YREB has relatively high disequilibrium. There are fewer cities in the high-value areas of carbon emissions, but the value of carbon emissions is relatively high. There is only one city in the world with an emission intensity of 87.44–174.13 kg/m^3^: Chongqing. Five cities have carbon emission intensities between 87.44 and 174.13 kg/m^3^, namely Ganzhou City, Huaihua City, Zunyi City, Qujing City, and Pu'er City. Except Chongqing, all the municipalities and provincial capitals in the YREB are located in the ow-value zone. The spatial distribution of carbon emissions mainly shows a "High west–Low east" pattern. The middle and high-value areas are primarily located in the upper and middle reaches of the Yangtze River. China's energy and mineral resources are distributed in regions with high carbon emissions, such as southern Hunan and southern Jiangxi. There are many places where coal is the primary energy source, including Guizhou, western Yunnan, and other regions. These regions should vigorously advocate using clean energy and promote energy-saving, environmentally friendly technologies in the subsequent development process to create a good low-carbon and environmental protection atmosphere in the whole society and improve the status quo of high carbon emissions.

#### Analysis of the spatial aggregation characteristics of carbon emissions in the YREB

**Figure 6:**
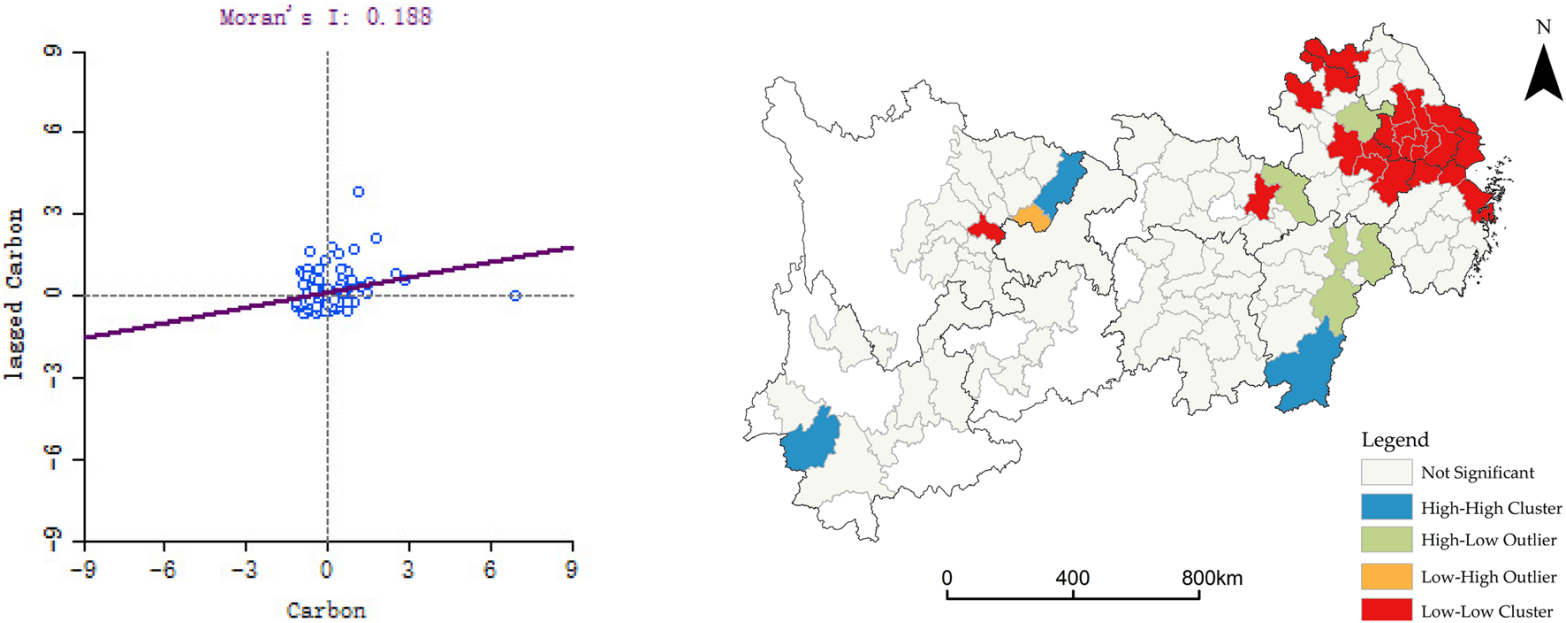
Spatial autocorrelation of carbon emissions.

The Global Morans *I* calculated by Geoda (https://geodacenter.github.io/download_windows.html) is 0.188. The p-value of 0.001. The Z-index test value is greater than 2.58. It shows that the YREB has significant spatial agglomeration characteristics for carbon emissions. Meanwhile, the Moran scatter plot and spatial distribution map demonstrate the correlation characteristics (Fig. 6). The first quadrant is the High-High agglomeration area, including Lincang, Dazhou, and Ganzhou, etc., indicating that these cities’ carbon emissions is high and the surrounding cities are also high. The second quadrant, Gangan, is Low–High agglomerations, indicating that there are high carbon emissions in cities surrounding these areas. The third quadrant is the Low-Low agglomeration area, including Shanghai, Nanjing, Suzhou, etc., indicating that these cities are low-value areas for carbon emissions. The fourth quadrant is the High-Low agglomeration area, including Hanggang, Fuzhou, Shangrao, etc., meaning that these cities have higher carbon emissions and are surrounded by cities with lower. The results show that the High-High concentration is in the western part of the YREB, and the Low-Low concentration is in the eastern part of the YREB, which is highly similar to the pattern of "High west–Low east".

The spatial distribution characteristics of the producer services show significant clustering. The wholesale and retail service industry has the highest degree of agglomeration. Financial and insurance industry agglomerations are concentrated in 10 central cities, such as Shanghai, Suzhou, and Wuxi. The main agglomeration centers of the real estate industry are in Shanghai, Hangzhou, Wuhan, and Chengdu. The main agglomeration centers of the leasing and business service industry are in Shanghai, Wuxi, Suzhou, and Hangzhou. The main agglomeration centers of scientific research and technology service industry are in 8 central cities, including Shanghai, Suzhou, and Nanjing. The main agglomeration centers of the wholesale and retail service industry are in Shanghai, Chongqing, and seven provincial capitals such as Nanjing and Hangzhou. The spatial distribution of carbon emissions mainly shows a "High west–Low east" pattern. The Emission intensity of carbon dioxide is not evenly distributed. Fewer cities are in high-value areas, but the value of carbon emissions is relatively high. All the municipalities and provincial capitals in the YREB, except Chongqing, are located in the low-value zone. It is noteworthy that the central city is the agglomeration center of the producer services and the low-value area of carbon emissions. This reflects that producer services and carbon emissions are spatially correlated, which sets the stage for the subsequent analysis.

### Spatial correlation analysis of producer services agglomeration and carbon emissions

This paper uses a Factor detector, Interaction detector, and Risk detector to verify the impact of producer services agglomerations on carbon emissions. Y is a dependent variable that measures carbon emissions. X_1_ is the number of POI in the financial and insurance industry (FI). X_2_ is the number of POI in the real estate industry (RE). In the leasing and business service industry (LB), X_3_ represents the number of POI. The number of POI in the scientific research and technical service industry (ST) is X_4_. X_5_ is the number of POI in the wholesale and retail service industry (WR). X_6_ is the number of POI in the transportation, storage, and postal industry (TS). Since the independent variables are numerical quantities, this paper uses ArcGIS 10.8 to discretize the independent variable X into six tiers based on the natural intermittent point hierarchy. GeoDetector (http://www.geodetector.org/) was used to calculate and analyze the grading results of the six indicators. The impact of agglomerating of different industries on carbon emissions is compared based on the results of the geographic detector.

#### Factor detection

Utilizing a Factor Detector to determine the extent to which each producer services explains carbon emissions spatial heterogeneity. The results of the factor detector are shown in Table 4. The q-values of the factor detection are as follows: wholesale and retail service industry (0.322) > finance and insurance industry (0.243) > transportation, storage and postal industry (0.238) > scientific research and technology service industry (0.233) > leasing and business service industry (0.204) > real estate industry (0.160). The nature of the producer services is different, and so is the impact on carbon emissions. The p-values are all less than 0.01. They indicate a statistically significant association between producer services agglomeration and carbon emissions. The largest q-value of the wholesale and retail service industry indicates that its agglomeration is the most important control factor of spatial heterogeneity. The agglomeration of the financial and insurance industry ranks second, indicating that its agglomeration also has a more significant impact on the spatial heterogeneity of carbon emissions. This is because the finance and insurance industry is more specialized and risky. It is an environment-friendly industry. Improving the development and scale of the financial and insurance industry contributes to reducing carbon emissions. The agglomeration of the wholesale and retail service industry has twice as much decisive power on the spatial heterogeneity of carbon emissions as the real estate industry. The real estate industry has the smallest q value, indicating it has the least influence, explaining 16% of the spatial differentiation. The q-values of other industries are in between, indicating that they also have an essential influence on the spatial heterogeneity of carbon emissions. Based on the above findings, it can be inferred that a practical and feasible solution to achieve carbon reduction targets without excessive impact on the economic is to regulate the agglomeration of the wholesale and retail industry as well as financial and insurance industry.

**Table 4:** Factor detection results.

Factor detection	*FI*	*RE*	*LB*	*ST*	*WR*	*TS*
q-value	0.243	0.160	0.204	0.233	0.322	0.238
p-value	0.000	0.000	0.000	0.000	0.000	0.000

#### Interaction detection

Exploring the influence of the two-pair synergistic agglomeration is using the interaction detector. The results are shown in Table 5. The interaction values of all combinations, such as "real estate industry-wholesale and retail service industry, leasing and business service industry-wholesale and retail service industry" are between the maximum value of two-factor q and the sum of two-factor q. They have a two-factor enhancement effect. This indicates that all interaction factors have a greater effect on the spatial differentiation of carbon emissions than any single factor. This is because the synergistic clustering of industries will increase the degree of industrial association, reducing wasted resources and decreasing transaction costs, thus improving economic and environmental efficiency. The interaction value of the "leasing and business service industry -wholesale and retail service industry" combination reaches 0.421, indicating that the "leasing and business service industry -wholesale and retail service industry" combination is the key interaction factor with the highest determination power on spatial heterogeneity. It is shown that the synergistic agglomeration of the leasing and business service industry and the wholesale and retail industry significantly affect the spatial pattern of carbon emissions.

**Table 5:** Interaction detection results.

Interaction factors	Interaction value	Interaction results
*FI* ∩ *RE*	0.309	Bifactor-enhanced
*FI* ∩ *LB*	0.284	Bifactor-enhanced
*FI* ∩ *ST*	0.279	Bifactor-enhanced
*FI* ∩ *WR*	0.393	Bifactor-enhanced
*FI* ∩ *TS*	0.293	Bifactor-enhanced
*RE* ∩ *LB*	0.253	Bifactor-enhanced
*RE* ∩ *ST*	0.287	Bifactor-enhanced
*RE* ∩ *WR*	0.419	Bifactor-enhanced
*RE* ∩ *TS*	0.318	Bifactor-enhanced
*LB* ∩ *ST*	0.296	Bifactor-enhanced
*LB* ∩ *WR*	0.421	Bifactor-enhanced
*LB* ∩ *TS*	0.275	Bifactor-enhanced
*ST* ∩ *WR*	0.411	Bifactor-enhanced
*ST* ∩ *TS*	0.262	Bifactor-enhanced
*WR* ∩ *TS*	0.417	Bifactor-enhanced

Many factors contribute to the status quo of carbon emissions along the YREB. Factor and interaction detection show that some factors that initially have weak explanatory power for the spatial heterogeneity will produce a two-factor enhancement when spatially superimposed with other elements. The combination of factors greatly enhances its ability to explain spatial differentiation in carbon emissions.

#### Risk detection

**Figure 7:**
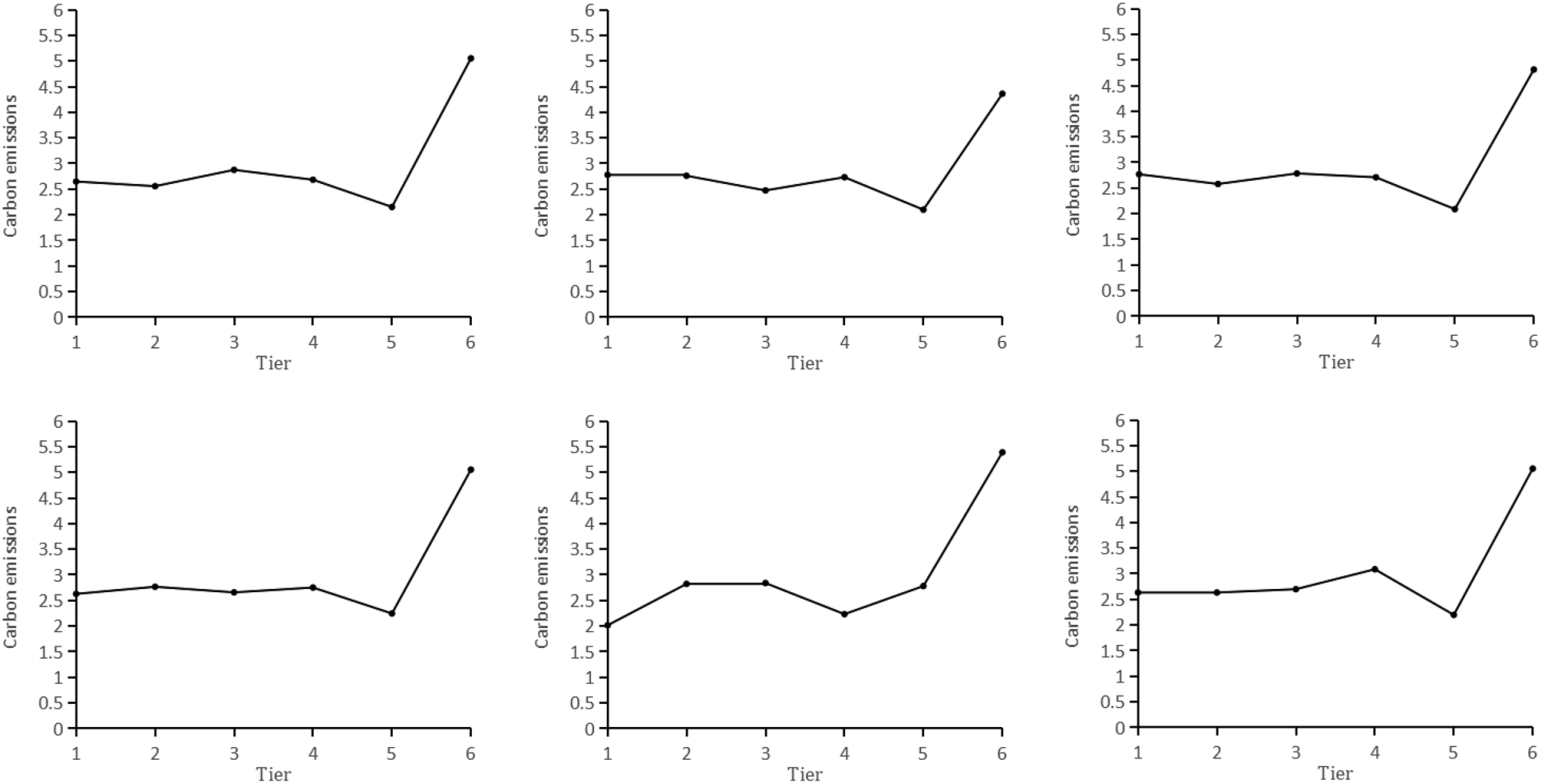
Risk detection results.

The results of the risk detector are shown in Fig. 7, which presents the influence trend of each producer services on carbon emissions. The horizontal coordinate represents the discrete concentration level of each sector in the producer services. The higher the class represents the higher concentration of the producer services. The vertical coordinate represents the average of the carbon emissions levels. Carbon emissions is linked to producer services. As the agglomeration of the financial and insurance industry, leasing and business service industry, transportation, storage, and postal industry have expanded, carbon emissions has increased, fallen, and then increased. Carbon emissions reaches the minimum level when the agglomeration reaches level 5. Carbon emissions peaks at the maximum power when the agglomeration comes level 6. With the agglomeration of the real estate industry, scientific research and technical service industry, carbon emissions shows a fluctuating trend. A peak in carbon emission intensity is reached at level 6 after the intensity bottoms out at level 5. As the wholesale and retail service industry agglomerate, carbon emissions rises, falls, and then rises again. Despite this, it is always higher than the carbon emissions of the first rank. Notably, the carbon emissions is the smallest when the concentration of industries other than the wholesale and retail service industry reaches the 5th level. It indicates that the concentration of producer services significantly inhibits carbon emissions to a certain extent. As a low-carbon industry, producer services agglomeration development will inevitably lead to improve of technological innovation and labor productivity in the agglomeration area. Within a certain agglomeration range, carbon emissions will reduce as producer services agglomeration increases.

## Discussion

In China, the government is highly motivated to develop producer services, particularly in terms of reducing carbon emissions. In the above context, analyzing the current situation of producer services is necessary. This paper systematically discusses the spatial distribution of various producer services agglomeration in the YREB under the carbon emissions reduction goal, which is helpful for readers to deeply understand the current situation of producer services agglomeration in the YREB. In terms of data selection, the paper does not select the panel data adopted by most scholars but focuses on POI and remote sensing data, providing a new data source for the research of industrial agglomeration. It is worth mentioning that the POI data has the characteristics of displaying the spatial information of the entity point elements of various social and economic sectors^64^. So this paper uses the Mean Nearest Neighbor Analysis to analyze whether the producer services agglomeration is clustered^65^, rather than the traditional methods such as the agglomeration index of the producer services agglomeration. This paper finds that the spatial distribution of producer services shows agglomeration characteristics, but the degree of agglomeration varies among different producer services. It confirms the findings of Du about the heterogeneity of producer services agglomeration^66^. This provides a factual basis for the follow-up study of the spatial correlation between producer services agglomeration and the carbon emissions in the YREB. Also, previous literature mostly use spatial econometric models when studying the relationship between industrial agglomeration and pollution, which better deal with spatial correlation and spatial spillover effects^67^. From the perspective of methodology, geographical detectors are introduced into this study. This method effectively reveals the spatial correlation between producer services agglomeration and carbon emissions from the perspective of spatial differentiation. It is found that there is industry heterogeneity. Factor detector explains that there is sectoral heterogeneity in the effect of producer services agglomeration on carbon intensity, a result corroborates Zhang’s findings^27^. The carbon emissions corresponds to the smallest value when five industries’ agglomeration, including the finance and insurance industry and the real estate industry, reach the 5th rank, and the smallest value when the agglomeration degree of wholesale and retail service industry reaches the 4th rank. It proves that producer services agglomeration has a suppressive effect on carbon emissions within a certain range. As industrial agglomeration increases, carbon emissions increases. When producer services agglomeration exceeds a certain threshold level, the carbon emissions increases. The result of producer services agglomeration will negatively affect carbon emissions^68^. In addition, the middle and high-value areas of carbon emissions are located in the upper and middle reaches of the Yangtze River. Compared with the middle and upper reaches, the producer services in the lower reaches exhibit significant carbon emissions reduction potential. The result proves the findings from a spatial perspective^69^. It proved to be of practical research significance in detecting the spatial association.

This study only explores the spatial correlation between producer services agglomeration and carbon emissions from the perspective of spatial distribution. Although the POI data is richer and more comprehensive than traditional data, its shortcomings as network data itself do not have authority cannot be ignored. Therefore, the subsequent research should fully explore the official data sources and combine them with mathematical measurements for in-depth investigation.

## Conclusion and policy recommendations

### Conclusion

This study establishes a mapping system from POI data to industry-type data and uses many methods to practically test the spatial correlation between producer services and carbon emissions in YREB with the starting point of industry spatial agglomeration characteristics. As a result of this study, new ideas are presented for promoting carbon emission reduction. Here are some of conclusions drawn from the study:

1. Producer services in the YREB exhibit significant spatial agglomeration characteristics. The financial and insurance industry is concentrated in the central cities. The real estate industry shows a radial expansion-type agglomeration pattern in space. The leasing and business service industry shows a concentrated cluster-type spatial distribution pattern. The scientific research and technology service industry shows a dispersed combination type spatial distribution pattern. The wholesale and retail industry shows a strip-like agglomeration pattern. The transportation, storage and postal industry shows a multi-center "core" agglomeration feature. On the whole, the provincial capitals and some central cities have become the preferred places for the layout of producer services. The distribution direction of each industry in the producer services is consistent with the regional shape of the YREB. The six major industries do not differ much in shape. All of them are in the southwest-northeast direction, which is closely related to the development axis of the YREB.
2. Carbon emissions exhibits precise spatial aggregation characteristics. The High-High concentration is in the western part of the YREB, and the Low-Low concentration is in the eastern part of the YREB, which is in accordance with the spatial distribution of carbon emissions. There are fewer cities in the high carbon emissions area, but the carbon emissions values are larger. The carbon emissions in the YREB is unbalanced.
3. The single factor detection results show that the concentration of the wholesale and retail service industry is the primary risk factor for the spatial differentiation of carbon emissions. Interaction detection results show that all factors’ explanatory power on spatial differentiation is higher than any single factor. The combination of "leasing and business service industry-wholesale and retail service industry" is the key interaction factor with the highest determining power. Although the real estate industry has the smallest q-value in factor detection, its combination with the remaining five factors all have a two-factor enhancement effect on carbon emissions.
4. Carbon emissions of the YREB shows a downward trend followed by an upward trend as producer services agglomeration increases. When five industries, including finance, insurance, and real estate aggregate to the fifth level, the carbon emissions is the smallest. Whenever the agglomeration degree of the wholesale and retail industry reaches level 4, the carbon emissions is the lowest. Specifically, producer services agglomeration can increase produce agglomeration effect and reduce carbon emissions within a certain range. Once producer services agglomeration exceeds a certain threshold level, the carbon emissions will increase. Producer services agglomeration will have a negative inhibitory influence on carbon emissions.

### Policy recommendations

Producer services development in the YREB has formed a distribution pattern of "central cities as nodes, multi-core to peripheral agglomeration and radiation". Based on the spatial correlation between producer services concentration and carbon emissions, it is particularly crucial to optimize producer services layout, modernize the service industry by speeding up its development, and further reduce carbon emission in the process of building the YREB into an eco-economic demonstration zone with high economic efficiency and good ecology.

1. Following the principle of ecological priority, plan to develop the producer services in the YREB from the top design. Considering the potential of urban green development in terms of resource endowment, talent base, industrial structure, etc., priority should be given to introducing of producer services that can help the transformation of regional high-polluting enterprises. Measures should be tailored to the specific needs of each region along the YREB. Under the "one-to-one" precise supporting development model, a distinctive supporting cluster of productive service industries will be formed.
2. Relying on the producer services specialized agglomeration, optimize the existing pattern of producer services. The central and western cities of the YREB have considerable environmental resource constraints. They should take the provincial capital and major cities as the benchmark, accelerate growth mode transformation, improve infrastructure and supporting services, and concentrate production factors to promote the producer services specialized agglomeration. Hubei, Hunan, and other transport hub areas can tap the advantages of a transport hub, commit to the development of intelligent transport to speed up the development of transport-related industries.
3. Relying on the producer services diversified agglomeration, promoting regional industries’ integrated development. The professional development of producer services in eastern cities is relatively mature, so industrial parks with leading industries and complete supporting facilities should be established to improve the diversified concentration level of producer services. Relying on the development advantages of urban agglomeration, build industrial clusters of producer services such as the Yangtze River Delta, form regional linkages, intensive and efficient, green and low-carbon industrial clusters. Shanghai, Jiangsu, Zhejiang, and other cities with a high degree of financial development should further gather high-quality resources at home and abroad to accelerate intelligent manufacturing. At the same time, they should actively build a highland for the diversified and integrated development of producer services and give full play to local effects.

## Data Availability

The datasets used during the current study available from the corresponding author on reasonable request.
